# Antimicrobial Peptides and Laying Hens Farming: A Review to Analyze the Improvement of the Animal Performance, Health, and Egg Quality

**DOI:** 10.1155/sci5/9748832

**Published:** 2025-09-22

**Authors:** Lorenza Brandão, Wendell Queiroz Leite, Mariane Severino, Thais Sevilhano, Danilo Florentino Pereira, Diogo Sartori, Eduardo Festozo Vicente

**Affiliations:** ^1^Graduate Program in Agribusiness and Development, School of Science and Engineering, São Paulo State University (UNESP), Tupã 17602-496, São Paulo, Brazil; ^2^Graduate Program in Animal Science, School of Agricultural and Veterinary Sciences, São Paulo State University (UNESP), Jaboticabal, São Paulo, Brazil; ^3^Department of Management, Development, and Technology, School of Sciences and Engineering, São Paulo State University (UNESP), Tupã 17602-496, São Paulo, Brazil; ^4^Department of Biosystems Engineering, School of Sciences and Engineering, São Paulo State University (UNESP), Tupã 17602-496, São Paulo, Brazil

**Keywords:** antimicrobial peptides, egg quality, immune system, laying hens

## Abstract

Microbial resistance is a global concern, potentially causing 10 million deaths by 2050 due to the ineffectiveness of conventional drugs. In this scenario, antimicrobial peptides (AMPs) emerge as a promising alternative, as they combat several pathogens without inducing resistance. AMPs stand out as a potential natural additive to replace antibiotics in laying hens, such as gentamicin and tetracycline, aiming for greater animal health. Therefore, this review aims to provide a general overview of layer poultry farming worldwide, especially in Brazil. Furthermore, the study brings data on the interaction of parameters of egg quality and blood cells and how AMPs can be beneficial and improve the laying hens' health.

## 1. Introduction

Egg production from laying hens faces complex challenges, especially in relation to sustainability and product quality. The growing demand for solutions that reduce environmental impacts while increasing productivity has driven innovations in the poultry sector [1]. Within this scenario, egg quality is a factor of central importance, being influenced by multiple intrinsic aspects, such as genetics, nutrition, and handling conditions of laying hens [2].

Recent research emphasizes the relevance of understanding the biological mechanisms that impact egg quality, such as the influence of bioactive compounds and the interaction of laying hens with the environment [3]. In this sense, technological advancement has become a priority in poultry farming, highlighting the minimization of conventional antibiotics, such as gentamicin and tetracycline, given the risk of antimicrobial resistance associated with their excessive employment [1].

In this concern, antimicrobial resistance is one of the most critical issues for public and animal health, being particularly challenging in intensive production systems, such as layer poultry farming. Strategies to mitigate this growing resistance have been widely discussed, including the use of antimicrobial peptides (AMPs) as safer and more effective alternatives to conventional antibiotics [4]. These peptides are designed to inhibit pathogens without harming host cells, representing a promising approach to health [5].

In this context, the adoption of more sustainable practices in poultry farming, such as reduced use of antibiotics and the implementation of alternatives such as AMPs, contributes to reducing the risk of antimicrobial resistance, a growing problem that affects both animal and human health. These reforms aim not only to improve productivity but also to ensure that practices in the poultry sector are in line with public health principles, minimizing negative impacts on the environment and consumer health [6].

In addition, improving animal welfare conditions in poultry sector reforms is also aligned with the concept of One Health. Promoting more natural production systems, with less environmental impact and better waste management, contributes to reducing the risk of zoonotic diseases. The incorporation of new feeding and disease control technologies, such as the use of AMPs, reflects this integration of the areas of animal, human, and environmental health, establishing a more comprehensive and sustainable approach [2].

The One Health concept seeks to integrate human, animal, and environmental health in a holistic approach, aiming to improve global public health. In the poultry sector, recent reforms have aligned with this concept by promoting practices that not only target bird health but also ensure food safety and environmental preservation. The integration of human, veterinary, and environmental health areas has proven crucial in the control of diseases transmissible between animals and humans, which is particularly relevant in layer hen farming, where management conditions directly influence public health [1].

Regarding the Sustainable Development Goals (SDGs), the use of AMPs is directly related to SDG 3 (Good Health and Well-being), as their use can reduce the impacts of antimicrobial resistance and improve the effectiveness of medical treatments. In addition, AMPs contribute to SDG 12 (Responsible Consumption and Production), offering a sustainable alternative to the overuse of antibiotics. Innovation in antimicrobial therapies also aligns with SDG 9 (Industry, Innovation and Infrastructure), promoting research and development of new therapeutic solutions and technologies in the field of public health [3].

Therefore, this review presents an overview of the interaction between the application of AMPs in layer hens and the physiological and environmental factors influencing egg production. It discusses, with emphasis, the underlying immune and microbiome mechanisms as well as hematological effects associated with AMPs use, highlighting their potential implications for improving animal health and egg quality and promoting sustainable practices in layer hen production.

## 2. Methodology

**Figure 1:**
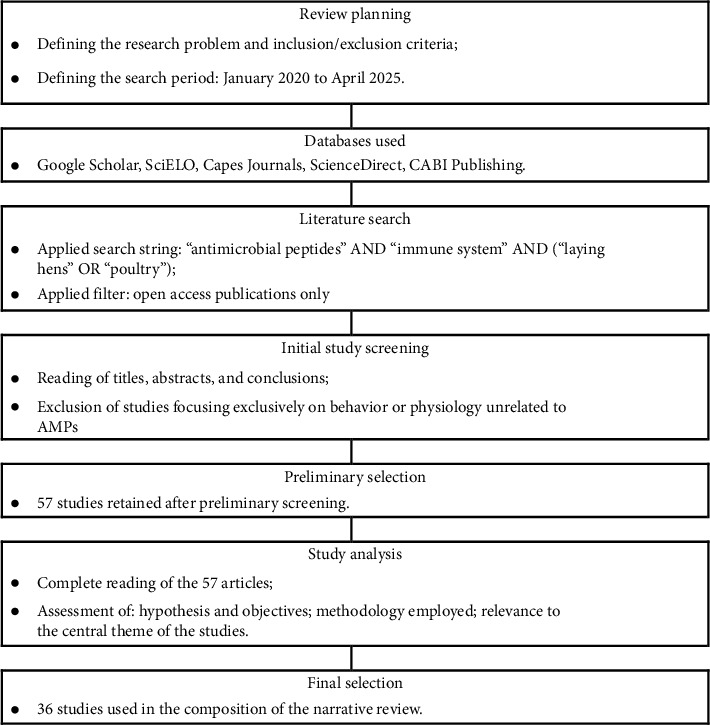
Flowchart of the methodology for the systematic literature review employed in this work.

Data collection was based on the analysis of narrative review studies, systematic reviews, or open access book chapters published between January 2020 and April 2025. During the initial search, a total of 135 files were found. After reading all abstracts, it was established that those studies that focused on analyses related to animal physiology and behavior were excluded. Therefore, a total of 120 documents were selected for the final review according to Figure 1.

The databases used were PubMed, ScienceDirect, and CABI Publishing. The string used during the search was “antimicrobial peptides” AND “immune system” AND (laying hens OR laying hens OR poultry). Files with the BibTex extension were downloaded and added to the “Rayyan” web application system.

Data analysis was initially based on reading the title, abstracts and conclusion of the 120 selected studies. The problem, hypothesis and objectives of the work were identified after a detailed reading, in addition to observing the references and relevance of the article for the study area. Finally, 36 documents were used to write this review.

## 3. Antimicrobial Peptides

The effectiveness of antimicrobials is not predictable against some emerging pathogens, which can cause undesirable side effects, in addition to microbial resistance. Therefore, the urgency for the development of new drugs is a reality, a fact already announced by the World Health Organization in 2015, which is putting at risk the fulfillment of the SDGs of the United Nations (UN) [7]. The National Action Plan for the Prevention and Control of Antimicrobial Resistance emphasizes the need for coordinated efforts to combat antimicrobial resistance, particularly in health and agriculture [7]. Similarly, environmental factors impact the physiological health of domestic poultry, which can influence their susceptibility to microbial infections [8].

In view of this, peptides are organic molecules composed of amino acids linked by a peptide bond and have several applications. Among them is a promising alternative to the use of conventional drugs for the eradication of diseases caused by fungi and bacteria. One class of peptides that combat these microorganisms is AMP molecules that are part of the innate defense system of most living organisms [9]. The understanding of disease mechanisms in poultry provides insights into microbial interactions, essential for advancing AMPs as therapeutic agents [10].

AMPs can be classified into five main groups: (1) peptides that adopt β-strand conformations, stabilized by two or three disulfide bonds; (2) peptides without cysteine (Cys) residues, which form amphipathic α-helical structures; (3) anionic peptides, rich in aspartate (Asp) and glutamate (Glu); (4) peptides rich in tryptophan (Trp), proline (Pro), and/or histidine (His) residues; and (5) peptides composed of rare amino acids, such as gramicidins, which consist of a polypeptide antibiotic obtained from *Bacillus brevis* [11]. These classifications offer valuable insights into designing new AMP-based drugs [3].

AMPs act by interacting with the plasma membrane of Gram-positive and Gram-negative bacteria, with the cell wall of Gram-positive bacteria being composed of a thick layer of peptidoglycans and lipoprotein acids, while the cell wall of Gram-negative bacteria is composed of the presence of an external membrane, presenting lipopolysaccharides (LPS) external to this membrane [12]. Thus, the selective interaction of the peptide with the membrane of the microorganism causes an extravasation of the intracellular fluid, causing its death [13].

Recent studies have also explored the application of AMPs in commercial laying hens, where they have shown promise in improving performance and physicochemical properties of egg quality by reducing microbial infections [6, 14, 15]. These findings further support the potential of AMPs as alternatives to traditional antimicrobial agents in layer hens' production [7, 15, 16].

Membranolytic AMPs act by interacting with the plasma membrane of Gram-positive and Gram-negative bacteria. Therefore, there is selectivity in the interaction of peptides with bacteria, causing extravasation of intracellular fluid leading to the pathogen's death. Basically, there are three ways of peptide-membrane interaction: barrel-stave pore, toroidal pore, or carpet. In the barrel-stave pore model, the helical peptides coat the inner layer of the pore, and the hydrophobic surfaces begin to interact with each other and with the phospholipid chains. For this, the length of the peptide must be sufficient to be able to cross the hydrophobic core of the plasma membrane. In the toroidal pore model, the lipid bilayer and the amphipathic helix of the peptide interact with each other to form a “channel” [17, 18]. The carpet model consists of the parallel agglomeration of peptides to the lipid bilayer of the plasma membrane, which forms a kind of carpet [19]. Electrostatic and hydrophobic interactions act together to promote bonds between lipids and peptides. Satisfactory results in combating microbial agents depend on these interactions.

Bioactive peptides, especially cationic ones, have a broad antimicrobial action, which makes them a topic of growing interest in the areas of biotechnology and medicine. Cationic peptides, such as those found in anurans, are widely studied for their ability to interact with lipid membranes and exert antimicrobial, antibiofilm, antifungal, and antiviral activities. These peptides are effective due to their positive charge, which facilitates interaction with the negative charges in the membranes of microbial cells, leading to their destabilization and, ultimately, cell death [4].

These peptides also have a mechanism of action that is different from conventional antibiotics, since they do not depend on specific resistance mechanisms, making them a promising alternative for combating resistant pathogens. When interacting with cell membranes, cationic peptides can create pores or channels, allowing the entry of toxic substances or the exit of essential components for the cell, which leads to the loss of cellular balance [12]. This action can be amplified by modifying their structures or by combining them with other substances, such as nanoparticles or polymers, which increases their therapeutic properties [20].

In addition, these peptides have been used in various areas, from veterinary medicine to the pharmaceutical industry, being explored in the treatment of microbial infections, especially those caused by resistant bacteria, such as those of the genus Salmonella [19]. The efficacy of cationic peptides is also being investigated to prevent infections in poultry, highlighting their potential to improve animal health and thus increase productivity in egg production systems [9].

**Figure 2:**
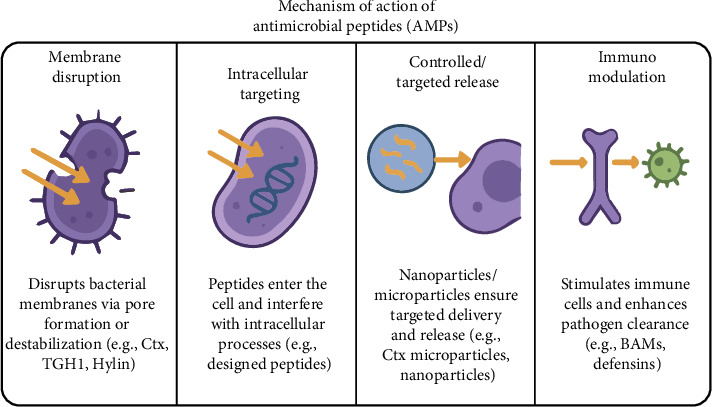
Main mechanisms of action and some examples of antimicrobial peptides related to their mode of action.

The use of AMPs, in addition to their ability to fight infections, is also an important strategy for sustainability, as it can reduce dependence on antibiotics and decrease the environmental impacts related to their excessive use of these drugs [21]. However, the development of therapeutic peptides needs to be careful, taking into account factors such as stability, toxicity and absorption of the compounds according to Figure 2 [22].

### 3.1. Use of AMPs in Layer Rearing

Laying hens farming has been modernizing globally, with an increasing emphasis on reducing antibiotic use, especially in response to antimicrobial resistance. Countries in the European Union and the United States have implemented strict policies to limit the use of antibiotics as growth promoters, promoting alternatives such as AMPs [19]. AMPs offer a promising solution, being effective in preventing and treating bacterial infections without the side effects associated with traditional antibiotics. Studies have shown that peptides such as Ctx(Ile^21^)-Ha, derived from the *Boana albopunctata* frog, show promising results, reducing mortality in laying hens and preventing bacterial load, especially in models of *Salmonella enteritidis* infection [20].

In Brazil, although there is an increasing effort to reduce antibiotic use, the country still faces significant challenges, such as the lack of effective alternatives challenges, including AMP stability, high production costs, potential toxicity, and the impact of economic and climate factors on layer hen farming [23]. However, research on the use of AMPs is advancing, and studies indicate that these compounds have the potential to improve bird health, egg quality, and productivity, especially under conditions of heat stress and infectious diseases. The adoption of AMPs in Brazil can also contribute to SDGs, such as SDG 3 (Good Health and Well-being) and SDG 12 (Responsible Consumption and Production), by reducing dependence on antibiotics and improving public health, in addition to promoting more sustainable and efficient production [14].

However, the use of AMPs in Brazil faces limitations, such as the need for a greater understanding of the mechanisms of action of peptides on the immune mechanisms of laying hens, especially in relation to the intestine and intestinal microbiota. Furthermore, the stability, production cost, and toxicity of AMPs are challenges that need to be overcome to enable their large-scale adoption [24]. Future studies should focus on improving these issues by exploring new forms of stabilization, such as nanoencapsulation, and performing more rigorous tests to establish the efficacy and safety of these compounds under different conditions [20].

Additionally, to increase the global relevance of this topic, it is important to compare studies conducted in Brazil with those conducted in other regions, such as Asia and Europe. This comparison can identify universal principles applicable to layer hen farming and strengthen the argument for the use of AMPs, in addition to promoting integration with SDGs, such as SDG 9 (Industry, Innovation and Infrastructure), encouraging innovation and research in new technologies [23, 25].

These discussions can be complemented with a change to more detailed analysis of the effects of AMPs on hematological parameters, such as calcium and hemoglobin levels, and on egg quality, addressing causal relationships more robustly, based on controlled experimental evidence [19].

### 3.2. Comparison Between Conventional Antibiotics and Antimicrobial Peptides in Layer Rearing

The growing antimicrobial resistance, exacerbated by the indiscriminate use of conventional antibiotics, has become a global public health problem [3]. Traditional antibiotics act by inhibiting essential cellular processes for bacteria, such as protein synthesis and DNA replication. However, prolonged and excessive use of these drugs has promoted the emergence of resistant strains, which compromises the effectiveness of treatments and increases the costs associated with public health [25].

In this context, AMPs emerge as a promising alternative to conventional antibiotics. These compounds, which can be of natural or synthetic origin, act in distinct ways, interacting with the cell membranes of pathogens or affecting essential intracellular mechanisms [24]. The main advantage of AMPs is their lower tendency to bacterial resistance, since they have multiple targets of action and their structures are more difficult for microorganisms to modify [25]. Furthermore, AMPs have broad action against a variety of pathogens, including bacteria, viruses, and fungi, which makes them an effective alternative for infection control [3].

However, despite their advantages, the use of AMPs faces significant challenges for their large-scale adoption. The production of these peptides in sufficient quantities and in an economical manner is still an obstacle, due to the high synthesis costs and the complexity of the biotechnological processes involved [25]. Furthermore, the stability of AMPs, which can be rapidly degraded in the biological environment, requires the development of delivery technologies, such as nanoencapsulation, to improve their efficacy and ensure their controlled release [24]. Other challenges include the assessment of the long-term safety of these compounds, especially in terms of toxicity and adverse effects, areas that still require further investigation [3].

### 3.3. Synergy of AMPs With Probiotics and Delivery Technologies (Encapsulation)

The synergy between AMPs and probiotics has been a growing focus of research in layer hen farming, especially with regard to improving gut health and increasing productivity. The combination of AMPs with probiotics can generate synergistic effects that strengthen the immune system of laying hens, promoting a balanced gut microbiome that is resistant to colonization by pathogens [26]. Probiotics help restore and maintain healthy gut flora, while AMPs directly combat pathogens, resulting in broader protection against diseases.

In addition, the use of technologies such as AMP encapsulation has shown promising results. Encapsulating AMPs in polymer microparticles can protect these peptides from degradation during the digestive process, ensuring their controlled release and effective action in the intestine of laying hens. This structural enhancement strategy not only increases the stability of AMPs but also allows these antimicrobial compounds to act more efficiently throughout the gastrointestinal tract of laying hens [20].

The combination of AMPs with encapsulation can also improve the bioavailability of these peptides, allowing their concentrations to reach more effective levels in the intestine, where they act to control pathogenic microorganisms. The use of polymer microparticles such as HPMCAS to encapsulate AMPs has a positive impact on the reduction of bacterial infections, such as those caused by Salmonella, without affecting the health of laying hens [11].

Therefore, the synergy between AMPs and probiotics, combined with structural improvement through encapsulation, represents an innovative approach to promoting intestinal health in laying hens. This combination offers a promising solution to reduce dependence on antibiotics in layer hen farming, while improving bird production performance. These technological innovations are essential for the advancement of modern layer hen farming, aligning with sustainability and public health trends [19].

### 3.4. Use of Antimicrobial Peptides in Laying Hens—Brazilian and Global Overview

Layer hen farming has undergone a global modernization process, with increasing emphasis on reducing antibiotic use, especially in response to antimicrobial resistance. Countries in the European Union and the United States have implemented strict policies to limit the use of antibiotics as growth promoters, encouraging alternatives such as AMPs [19]. AMPs offer a promising solution, as they are effective in preventing and treating bacterial infections without the side effects associated with traditional antibiotics. Research has indicated that peptides such as Ctx(Ile^21^)-Ha can reduce bird mortality and bacterial load, especially in models of *S. enteritidis* infection [20].

In Brazil, although there is a growing movement to reduce antibiotic use, the country still faces significant challenges, such as the lack of effective alternatives and the impact of economic and climate factors on layer hens' production [23]. However, research on the use of AMPs is advancing, and studies indicate that these compounds have the potential to improve bird health, egg quality, and productivity, especially under conditions of heat stress and infectious diseases. AMPs could also help Brazil move toward SDGs, such as SDG 3 (Good Health and Well-being) and SDG 12 (Responsible Consumption and Production), by reducing antibiotic use, improving public health, and supporting sustainable production practices [14].

However, the use of AMPs in Brazil still faces limitations, such as the need for a deeper understanding of how peptides influence the immune mechanisms of birds, especially in relation to intestinal health and microbiota balance. In addition, challenges such as AMP stability, production cost, and potential toxicity need to be overcome to make their large-scale adoption viable [24]. More research is needed to address these challenges, investigate new stabilization techniques, such as nanoencapsulation, and conduct more detailed tests to confirm the efficacy and safety of these compounds [20].

In addition, to increase the global relevance of this topic, it is important to compare studies conducted in Brazil with those from other regions, such as Asia and Europe. Comparing studies across regions can reveal common principles for layer hen farming and support the adoption of AMPs, as well as promote innovation and research, in line with SDG 9 (Industry, Innovation and Infrastructure) [23, 25]. These discussions can be complemented with an analysis of the effects of AMPs on hematological parameters—such as calcium and hemoglobin levels—and egg quality, exploring causal relationships more robustly through controlled experimental evidence [19].

### 3.5. Effects of AMPs on Mortality, Hematological Parameters, Productivity, and Egg Quality

Egg quality is measured by density or specific gravity, shell strength and thickness, egg weight, albumen height, and Haugh unit (HU) [27].

The density of eggs can be measured by immersing them in saline solutions ranging from 1060 to 1100 g/cm^3^. Specific gravity is the value of the lowest density at which the egg floats. If the egg does not float, you must dip it in the next solution, which contains more salt. The appropriate value for this parameter is greater than 1085 g/cm^3^, as the greater the specific mass, the greater the eggshell density and, consequently, the greater the resistance [28].

The thickness of the eggshell is evaluated using a micrometer, where three equidistant points are listed in the equatorial zone of the eggshell, and after measurements and calculation of the arithmetic mean between the three values, the thickness is [16]. It is expected that the value is above 0.35 mm for the thickness to be considered adequate [29]. There is a relationship between the weight of the egg and the resistance of the shell. These two quantities are generally inversely proportional [16].

Therefore, ideal egg weight is a fundamental parameter in evaluating the quality of eggs produced by commercial laying hens. The ideal weight for eggs from commercial chickens varies between 55 and 65 g, depending on the bird's strain and stage of production. This value is related to factors such as nutrition, genetics, and management adopted, being one of the main indicators in the classification of eggs [30]. Adequate weight ensures a balanced proportion between yolk and white, which contributes to good physical and chemical quality of the product. Furthermore, controlling egg weight can prevent structural defects, such as cracks and deformations, improving the appearance and commercial value of the product [16].

As for the height of the albumen, it is another important aspect for the internal quality of the egg, reflecting its freshness and integrity. The ideal albumen height should be at least 6 mm, which indicates a fresh egg with a firm and well-formed white [7]. When this height decreases, it may be a sign of egg aging or stress conditions in the laying hens. Factors such as temperature, lighting, and feed quality directly affect albumen height and, consequently, egg quality. Maintaining an adequate height of the albumen is essential to preserve the sensorial characteristics of the egg, such as the consistency of the white and the ease of breaking the shell [31].

The HU is the measurement of the height of the albumen corrected by the weight of the egg and must be above 72 for the egg to be considered of excellent quality based on this parameter. Values between 60 and 73 indicate that the egg has high quality, and values below 60 indicate low quality [33]. Therefore, Table 1 below illustrates the ideal parameters for egg quality in the review.

The egg analysis time has a direct impact on the evaluation of several quality parameters, such as density, resistance, shell thickness, egg weight, albumen height, and HU. The density of the egg, which must be greater than 1.085 g/cm^3^, can be altered by inadequate storage conditions or by analyses carried out after long periods of time [28]. During storage, the egg tends to lose mass, which can reduce its density and, consequently, its quality. The resistance of the shell, which ideally should be 4 kgf, is also sensitive to the analysis time, as the shell can undergo changes in its integrity with aging, becoming more fragile [16].

Shell thickness is another relevant parameter, with the ideal value being greater than 0.35 mm [29]. Over time, the shell can become thinner due to the loss of calcium, which reduces the physical resistance of the egg. The height of the albumen, which must be at least 6 mm, also decreases as the egg ages [7, 16].

This occurs because the albumen loses water and its proteins change, impairing the assessment of the egg's freshness.

The analysis time directly influences this measurement, as the albumen becomes thinner as the egg ages. Therefore, if the analysis is not carried out quickly after laying hens, the HU values may be compromised, resulting in a less accurate assessment of egg quality.

The weight of the egg, which should vary between 55 and 65 g, can also be altered by factors such as bird feeding and storage time [30, 31]. The egg loses weight over time, which can reduce the recorded value and make it difficult to assess its nutritional value and quality. The ideal values found in the literature are in Table 1.

In summary, the time between laying hens and analysis is essential to ensure accuracy in evaluating egg quality parameters. Quality maintenance is directly related to the storage time and handling conditions of the egg until the moment of analysis. Extending this period may negatively affect the assessment of ideal parameters and, consequently, the classification of the final product.

In addition to the antimicrobial and immune system modulating effects, AMPs also have significant impacts on hematological parameters and egg quality in laying hens. These effects are mainly due to the ability of AMPs to control intestinal infections, reduce systemic inflammatory processes, and improve the absorption of nutrients essential for blood formation and egg production [14, 34].

The modulation of the intestinal microbiome promoted by AMPs increases beneficial microorganisms and reduces the pathogen population, resulting in a lower release of LPS and endotoxins into the bloodstream [3]. This control reduces the activation of inflammatory cytokines, which helps stabilize the leukogram and prevents changes in hematological indicators such as mean corpuscular volume (MCV) and red blood cell distribution width (RDW) [9].

Regarding egg quality, the benefits extend to shell formation and internal content. The reduced exposure of laying hens to bacterial toxins contributes to the preservation of the albumen and cascade structure, reducing losses due to cracks and improving egg conservation [24, 25]. This occurs, in part, by improving intestinal calcium absorption and the efficient allocation of energy for productive functions, since the body is not overloaded by inflammatory electrical processes [14, 34].

The use of technologies such as encapsulation of AMPs has also shown promising results. The controlled release of encapsulated peptides in the intestine favors local action against pathogens, reducing infections such as those caused by Salmonella and contributing to the production of safer eggs with lower commercial losses [19, 20].

In addition, when used in combination with probiotics, AMPs can enhance local and systemic immune responses. This combination favors the production of immunoglobulin A (IgA) in the gastrointestinal tract and improves nutrient absorption, which is reflected in more balanced hematological parameters, as well as eggs with greater weight, thicker shells, and less variation between batches [15, 26].

Therefore, the effects of AMPs are not limited to their antimicrobial function; they directly influence hematological physiology and egg production, reinforcing their potential as a functional additive in modern layer hen farming [30, 35].

Recent studies have investigated the effectiveness of AMPs in reducing mortality and increasing productivity of laying hens. A meta-analysis revealed that the use of AMPs in layer hens' feeds can significantly reduce mortality, especially under conditions of *Salmonella* infection. These results indicate that, in addition to their antimicrobial properties, AMPs can play a crucial role in improving the overall health of laying hens, consequently positively impacting the survival rate [19].

The use of AMPs is also associated with increased egg production, mainly due to their action in promoting a healthy intestinal environment. AMPs improve nutrient absorption by reducing intestinal infections and promoting a balanced microbiome. These factors contribute to an increase in feed efficiency, resulting in greater productivity and less waste of resources [36].

The effect of probiotic microorganisms combined with AMPs in laying hens showed a significant increase in the egg production rate, associated with a decrease in intestinal diseases and improvement in hematological parameters [15]. The reduction in mortality and increased productivity are attributed to the synergistic effect of AMPs with the intestinal microbiota, which results in a more efficient digestive system and better immune health of the laying hens.

In a systematic review carried out by Silveira and collaborators [14], the results indicate that the administration of AMPs is especially effective in contexts where laying hens are exposed to stressful conditions, such as bacterial infections and high population densities. The application of AMPs in situations such as these not only improves bird health but also contributes to the improvement of production indicators, including egg quality and growth performance. These findings suggest that AMPs have great potential as feed additives for laying hens, with a direct impact on productivity and reduced mortality.  Table 2 shows the main AMPs applied in laying hens, and Table 3 shows the cause and effect relationships between the mechanisms of action of AMPs on hematological and egg quality parameters.

### 3.6. Interaction of AMPs With the Immune System and Gut Microbiome of Laying Hens

AMPs play an important role in modulating the immune system of laying hens, interacting directly with immune cells and influencing the intestinal microbiome. AMPs can exert their antimicrobial functions through two main mechanisms: destabilization of the cell membrane of pathogens and intracellular interaction, affecting essential metabolic processes. These mechanisms allow AMPs to attenuate bacterial infections, which results in a more effective immune response [3].

In addition, studies indicate that AMPs can influence the microbial diversity of the intestine of laying hens, promoting the maintenance of a healthy microbiome [9]. By interacting with cells of the intestinal immune system, such as enterocytes, AMPs can stimulate the release of cytokines and other bioactive molecules that strengthen the immune response. These effects are highly relevant in the context of layer hens' intestinal health, since a balanced microbiome contributes to disease prevention and optimization of productive performance.

The use of AMPs can modulate intestinal inflammation and reduce pathogen load, facilitating the recovery of laying hens from bacterial infections [37]. The interaction between AMPs and the avian immune system is not limited to the inflammatory response; these peptides also play a crucial role in regulating the balance of the intestinal microbiome, favoring the growth of beneficial microorganisms while inhibiting harmful pathogens.

Thus, AMPs can act as a promising alternative in the control of enteric diseases and in the promotion of intestinal health. AMPs also directly affect cells of the immune system, such as macrophages and lymphocytes, modulating their activities [36]. By interacting with these cells, AMPs can alter the immune responses of laying hens, making them more efficient in defending against infections.

**Figure 3:**
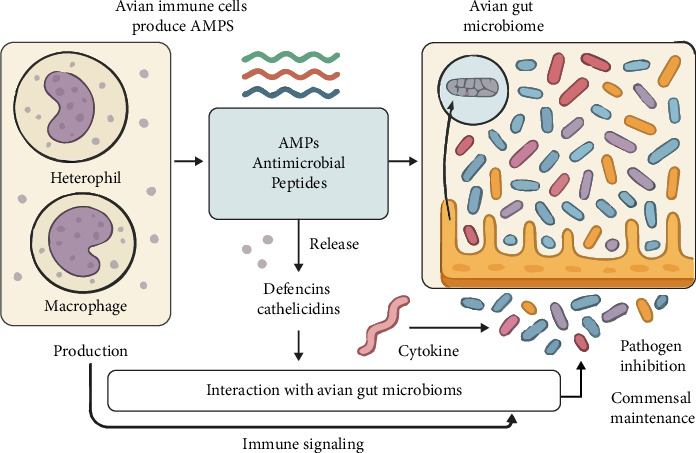
Interaction of AMPs with the avian immune system and gut microbiome. Source: adapted from Brasil [1], Benfield and Henriques [3], He and Deber [9], Espeche et al. [12], Silveira et al. [14], Yang et al. [17], Roque-Borda et al. [19], Roque-Borda et al. [20], Talapko et al. [24], Ma et al. [37], Zhao et al. [34], and Dehsahraee et al. [26].

Therefore, the use of AMPs in layer hen farming not only strengthens laying hens' defense against pathogens but also improves the overall immune capacity of laying hens.  Figure 3 below illustrates the interaction of AMPs with the avian immune system and intestinal microbiome.

By investing in technologies that promote bird health in a more efficient and less harmful way to the environment, layer hen farming contributes directly to the One Health model. The implementation of management strategies that include the use of probiotics and AMPs to prevent enteric diseases in laying hens represents an important innovation, since it not only favors the health of animals but also reduces the use of antibiotics, promoting a more sustainable and safe production for human health [2].

## 4. Final Considerations

AMPs emerge as a promising alternative to address the problems caused by antimicrobial resistance, one of the biggest challenges for global public health. Their ability to combat a wide variety of pathogens, including bacteria resistant to conventional antibiotics, makes them an important option in infection control, both in hospitals and industry. Furthermore, the action of AMPs is not limited to treating infections but can also promote intestinal health and strengthen the immune system, especially in the layer hen farming context.

The use of peptides as additives in layer hens' feed has shown positive results, such as reducing mortality, controlling microbial infections, and increasing egg production. The ability to modify the structure of peptides and combine them with other substances, such as nanoparticles, can also improve their therapeutic properties, making them more effective in combating diseases and promoting health.

The process involves some technical challenges; the purification, characterization, and microencapsulation steps guarantee the quality and stability of the peptides, making them more effective for different scientific and medical applications.

In summary, the advancement of studies and innovations in this area is essential to consolidate AMPs as a safe and effective solution to combat infections, representing a viable alternative in the face of growing resistance to antibiotics.

## Figures and Tables

**Table 1:** Ideal parameters for egg quality.

Parameters	Optimal value	Authors
Density	> 1085 g/cm^3^	Johnson and Merritt [28]
Resistance	4 kgf	Kocevski et al. [16]
Shell thickness	> 0.35 mm	Vaccinar [29]
Egg weight	55–65 g	Camargo et al. [30]; Oliveira et al. [31]
Albumen height	≥ 6 mm	Oliveira Castro et al. [32]
Haugh unit (HU)	> 72	Romero et al. [33]

**Table 2:** Main antimicrobial peptides (AMPs) applied in laying hens.

Peptide	Source	Activity	Mechanism of action	Authors
Ctx(Ile^21^)-Ha	Animal: frog (*Boana albopunctata*)	Antimicrobial	Interacts with the cell membrane of bacteria, causing porosity and disintegration	Benfield and Henriques [3]; Roque-Borda et al. [20]
Ctx(Ile^21^)-Ha in microparticles	Animal: frog (*B. albopunctata*)	Antimicrobial (oral use in laying hens)	Enteric release, interaction with bacterial cell wall	Roque-Borda et al. [19]
Hylin A1, Hylin A2, Hylin A3	Animal: frog (*Boana pulchella*)	Antimicrobial	Penetration and destabilization of the lipid bilayer	Aguilar et al. [4]
TGH1	Animal: Mollusc (*Tegillarca granosa*)	Antimicrobial (acts against *Vibrio parahaemolyticus*)	Pore formation and destruction of the bacterial membrane	Yang et al. [17]
Designed peptides (various)	Synthetic	Antimicrobial and anticancer	Multiple mechanisms: membrane insertion, aggregation, intracellular action	Benfield and Henriques [3]; Zhang et al. [25]; Talapko et al. [24]
Various (in nanoparticles)	Synthetic or Animal	Antimicrobial (including against Mycobacterium *tuberculosis*)	Membrane interaction and controlled release by nanoparticles	Primo et al. [11]
Various (summary of mechanisms)	Various	Antimicrobial, immunomodulatory, and anticancer	It varies between membrane rupture, pore formation, translocation, and intracellular inhibition	Zhang et al. [25]; Talapko et al. [24]; Espeche et al. [12]; Ma et al. [37]
Defensins	Animal: Humans (*Homo sapiens*), Laying hens (*Gallus gallus domesticus*)	Antimicrobial, antivirus, and anticancer	Interacts with the cell membranes of pathogens, causing direct damage and activating the immune response	Ferreira et al. [23]; Silveira et al. [14]
Cathelicidins	Animal: Cattle (*Bos taurus*)*; P*igs (*Sus scrofa*); and Laying hens (*Gallus gallusdomesticus*)	Antimicrobial and antiviral	They inhibit the cell wall of pathogens, promoting their destruction	Roque-Borda et al. [19]
Bovine Adrenal Medulla peptides (BAMs)	Animal: Cattle (*Bos taurus*)	Antimicrobial antifungal	They act to modulate the immune response, activating immune system cells and altering bacterial membranes	Silveira et al. [14]; Roque-Borda et al. [19]

**Table 3:** Cause and effect relationships between mechanisms of action of AMPs on hematological and egg quality parameters.

Mechanism of action of PAMs	Effects on the blood (hematological parameters)	Effects on the egg quality	Authors
Disruption of membranes of pathogenic bacteria, causing cell lysis	Reduction of systemic inflammation; improvement in leukogram with reduced leukocytosis	Reduction of systemic microbial contamination; improvement in albumen pH and viscosity	Benfield and Henriques [3]; Espeche et al. [12]
Inhibition of bacterial DNA, RNA, and protein synthesis	Less endotoxin release, greater blood count stability, and less immunological stress	Preservation of the albumen structure and shell integrity	Zhang et al. [25]; Talapko et al. [24]
Modulation of the intestinal microbiome, favoring beneficial bacteria	Increased lymphocyte and monocyte activity; strengthening of the innate immune response	Greater calcium deposition in the shell; eggs with more uniform thickness	Zhao et al. [34]; Silveira et al. [14]
Use of encapsulated AMPs, with targeted release in the intestine	Reduced mortality and less activation of hematological compensation mechanisms	Decreased incidence of Salmonella; eggs with better sanitary quality	Roque-Borda et al. [19]; Roque-Borda et al. [20]
Neutralization of lipopolysaccharides and other bacterial PAMPs	Less activation of pro-inflammatory cytokines; stability in RDW and MCV values	Laying stabilization; improvement in albumen density	He and Deber [9]; Rodrigues et al. [35]
Combined action with probiotics and single-cell proteins	Greater production of IgA and intestinal immune cells; strengthening of the immune barrier	Increased egg weight and mass; less variation in quality between batches	Dehsahraee et al. [26]; Sjofjan et al. [27]
Regulation of the intestinal microbiota and intestinal permeability	Better absorption of nutrients such as iron and amino acids; increased hematocrit and hemoglobin	Thicker shell and lower breakage rate	Zhao et al. [34]; Camargo et al. [30]

## Data Availability

Data sharing is not applicable to this study as no datasets were generated or analyzed during the current study.
